# Dimerization of the pulmonary surfactant protein C in a membrane environment

**DOI:** 10.1371/journal.pone.0267155

**Published:** 2022-04-27

**Authors:** Hanna Korolainen, Fabio Lolicato, Giray Enkavi, Jesús Pérez-Gil, Waldemar Kulig, Ilpo Vattulainen

**Affiliations:** 1 Department of Physics, University of Helsinki, Helsinki, Finland; 2 Heidelberg University Biochemistry Center, Heidelberg, Germany; 3 Faculty of Biology, Department of Biochemistry and Molecular Biology, Research Institute “Hospital 12 de Octubre (imas12)”, Complutense University, Madrid, Spain; Universidade Nova de Lisboa Instituto de Tecnologia Quimica e Biologica, PORTUGAL

## Abstract

Surfactant protein C (SP-C) has several functions in pulmonary surfactant. These include the transfer of lipids between different membrane structures, a role in surfactant recycling and homeostasis, and involvement in modulation of the innate defense system. Despite these important functions, the structures of functional SP-C complexes have remained unclear. SP-C is known to exist as a primarily α-helical structure with an apparently unstructured N-terminal region, yet there is recent evidence that the functions of SP-C could be associated with the formation of SP-C dimers and higher oligomers. In this work, we used molecular dynamics simulations, two-dimensional umbrella sampling, and well-tempered metadynamics to study the details of SP-C dimerization. The results suggest that SP-C dimerizes in pulmonary surfactant membranes, forming dimers of different topologies. The simulations identified a dimerization motif region V^21^xxxVxxxGxxxM^33^ that is much larger than the putative A^30^xxxG^34^ motif that is commonly assumed to control the dimerization of some α-helical transmembrane domains. The results provide a stronger basis for elucidating how SP-C functions in concert with other surfactant proteins.

## Introduction

During breathing, air is taken into human lungs, where it travels through the trachea to the left and right bronchi, and eventually ends up in small sacks called alveoli. Human lungs have roughly 700 million alveoli, which makes up to 70 m^2^ of active surface enabling the gas exchange [1].

Pulmonary surfactant, lining the alveoli, is a complex substance consisting of phospholipids and proteins [2]. The main function of pulmonary surfactant is to reduce the surface tension of fluids inside the alveoli. Pulmonary surfactant undergoes continuous compression-expansion cycles, as the gas exchange takes place. The volume difference in the lungs between inhalation and exhalation is about 10% [3]. To minimize the volume change, pulmonary surfactant must maintain a low surface tension inside the alveoli. The reduction of the surface tension by the surfactant decreases the pressure needed for alveoli expansion during inhalation and prevents the alveoli from collapsing during exhalation. In addition to the reduction of the surface tension, pulmonary surfactant is also an important barrier against pathogens, and thus it is an essential part of the immune defense. Pulmonary surfactant also provides mechanical stability during the expansion-compression breathing cycles [4].

Pulmonary surfactant is a multilayered substance of lipids, composed of both monolayer and bilayer structures [4]. Monolayers are located nearest to the air-liquid interface, whereas various kinds of membrane structures are found near and further from the air-phase. Different events, such as aggregation, exchange, and mixing and leaking of the lipid content, take place in these membrane structures [3]. Around 90 mass-% of pulmonary surfactant is composed of lipids, of which phosphatidylcholine (PC) and in particular 1,2-dihexadecanoyl-*sn*-glycero-3-phosphocholine (DPPC) is the most abundant, complemented by cholesterol, whose role and effect in pulmonary surfactant is also significant. The other phospholipids include phosphatidylglycerol (PG), phosphatidylinositol (PI), phosphatidylethanolamine (PE), sphingomyelin, and lysoPC [3,5,6].

Approximately 8–10% of the surfactant mass consists of surfactant proteins (SPs), mainly surfactant proteins A, B, C, and D (SP-A, SP-B, SP-C, and SP-D, respectively). SP-A and SP-D are involved in the innate immune system, while SP-B and SP-C aid the function of pulmonary surfactant by modulating its behavior at the air-liquid interface during breathing [7,8]. SP-B and SP-C are relatively small and hydrophobic surfactant proteins found in different lipid layers [4,9,10].

Main functions of SP-C include assisting in the transfer of lipids between the monolayers and the multilayered structures below the air-liquid interface, enhancing the adsorption of surface-active molecules into the air-liquid interface, and maintaining the integrity of the multilayered structure [3,4]. It may also have a role in the immune defense along with SP-A and SP-D, and it may regulate the pressure changes in the bilayers [11]. While the lack of SP-C does not lead to immediate death, it is essential for sustaining the long-term respiratory dynamics [12]. There are data suggesting that SP-C may also mediate internalization and recycling of spent/inactivated surfactant structures by pneumocytes and macrophages [3,13]. Given this, one can conclude that SP-B and SP-C are proteins that are crucial for survival. Further, deficiency of SP-C leads to severe chronic respiratory pathologies, such as idiopathic pulmonary fibrosis (IPF) and interstitial lung disease (ILD) [14–19]. Misfolding and aggregation of SP-C, on the other hand, are associated with pulmonary alveolar proteinosis [20].

**Fig 1:**
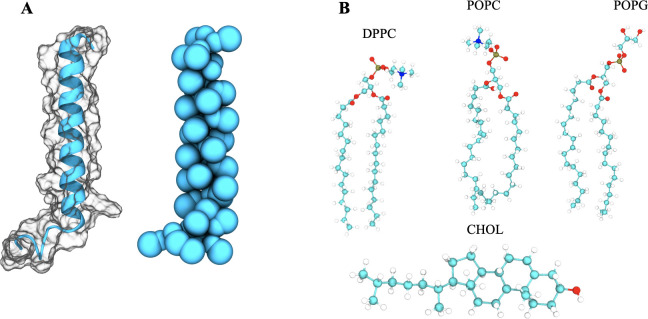
A) Atomistic (left) and coarse-grained (right) model of surfactant protein C (SP-C). B) Chemical structure of lipids used to mimic the pulmonary surfactant membrane: DPPC, POPC, POPG, and cholesterol (CHOL). See the text for details.

The biological function of SP-C is linked to its structure. With only 35 amino acids, the hydrophobic SP-C, shown in Fig 1A, is the smallest of the four surfactant proteins. It has a primarily α-helical structure with an in principle unstructured N-terminal region. The α-helix of SP-C is composed mainly of valine residues, which is unusual, since valines are typically found in β-sheets, rather than α-helices [21]. Also, there is reason to stress the importance of the presence of a chaperon-like BRICHOS domain at the precursor of SP-C, which likely assists the proper folding of the mature protein [22]. The C-terminal helical region of SP-C contains a region that is strictly conserved among all mammals, called the A^30^LLMG^34^ motif. Based on its similarity to the GxxxG motif found in the glycophorin A, this motif has been hypothesized to be a dimerization interface [23–25]. Very recently, Barriga and coworkers [26] suggested that the dimerization of SP-C could trigger budding and nano-vesicularization of pulmonary surfactant membranes.

In this work, we used biomolecular simulations through molecular dynamics (MD) simulations, two-dimensional umbrella sampling, and well-tempered metadynamics to study the details of SP-C dimerization. Our results reveal not only the mechanism and energetics of SP-C dimerization in the lipid bilayers, but also the various energetically favorable dimer configurations. Interestingly, we found that a region much larger than the putative A^30^xxxG^34^ motif is involved in the dimerization: the V^21^xxxVxxxGxxxM^33^ motif.

## Methods

### Coarse-grained systems with two SP-C monomers embedded into a lipid bilayer

The structure of the SP-C protein (Fig 1A) was obtained from Protein Data Bank (conformer 1 from PDB ID: 1SPF [27]). In this work, SP-C proteins were explored without palmitoylation. The effects of palmitoylation will be investigated in future work. The coarse-grained (CG) MARTINI force field model for SP-C was prepared using the *martinize*.*py* script [28–30]. A lipid bilayer containing 50 mol% DPPC (1,2-dihexadecanoyl-*sn*-glycero-3-phosphocholine), 25 mol% POPC (1-palmitoyl-2-oleoyl-*sn*-glycero-3-phosphocholine), 15 mol% POPG (1-palmitoyl-2-oleoyl-*sn*-glycero-3-(phospho-rac-(1-glycerol))), and 10 mol% cholesterol ((3β)-cholest-5-en-3-ol; CHOL) [3] (see Fig 1B) was built using Packmol [31]. Two SP-C monomers were then inserted into the bilayer, such that they were 4.5 nm apart from each other, using the method described by Javanainen [32]. The bilayer with two embedded proteins was subsequently solvated and an appropriate number of counterions (sodium and chloride) was added to neutralize the system. The initial dimensions of the system were 8 nm × 8 nm × 10 nm. Detailed compositions of the simulated systems are given in Table 1.

**Table 1:** Detailed composition of the systems studied in this work.

System type	SP-C	DPPC	POPC	POPG	CHOL	Water	Na^+^	Cl^-^	Number of simulations
**Free energy calculations (CG)**	2	300	150	90	60	7500	84	0	1393 (each 4 μs long)
**Unbiased simulations (AA)**	2	64	32	20	12	6394	20	6	18 (each 1 μs long)

### Free energy calculations of dimerization

The above-discussed systems were subsequently energy minimized and equilibrated, after which free energy calculations of dimerization were carried out. The simulation time step was set to 20 fs. The Verlet cut-off scheme was used for neighbor searching, and the neighbor list was updated every 10 steps. Electrostatic interactions were treated using the reaction-field algorithm [29]. The radius for van der Waals interactions was set to 1.1 nm. A constant temperature of 310 K was kept using the v-rescale thermostat with a coupling constant of 1 ps [33]. Pressure was maintained semi-isotropically at 1 bar using the Parrinello-Rahman barostat with a coupling constant of 12 ps [34].

**Fig 2:**
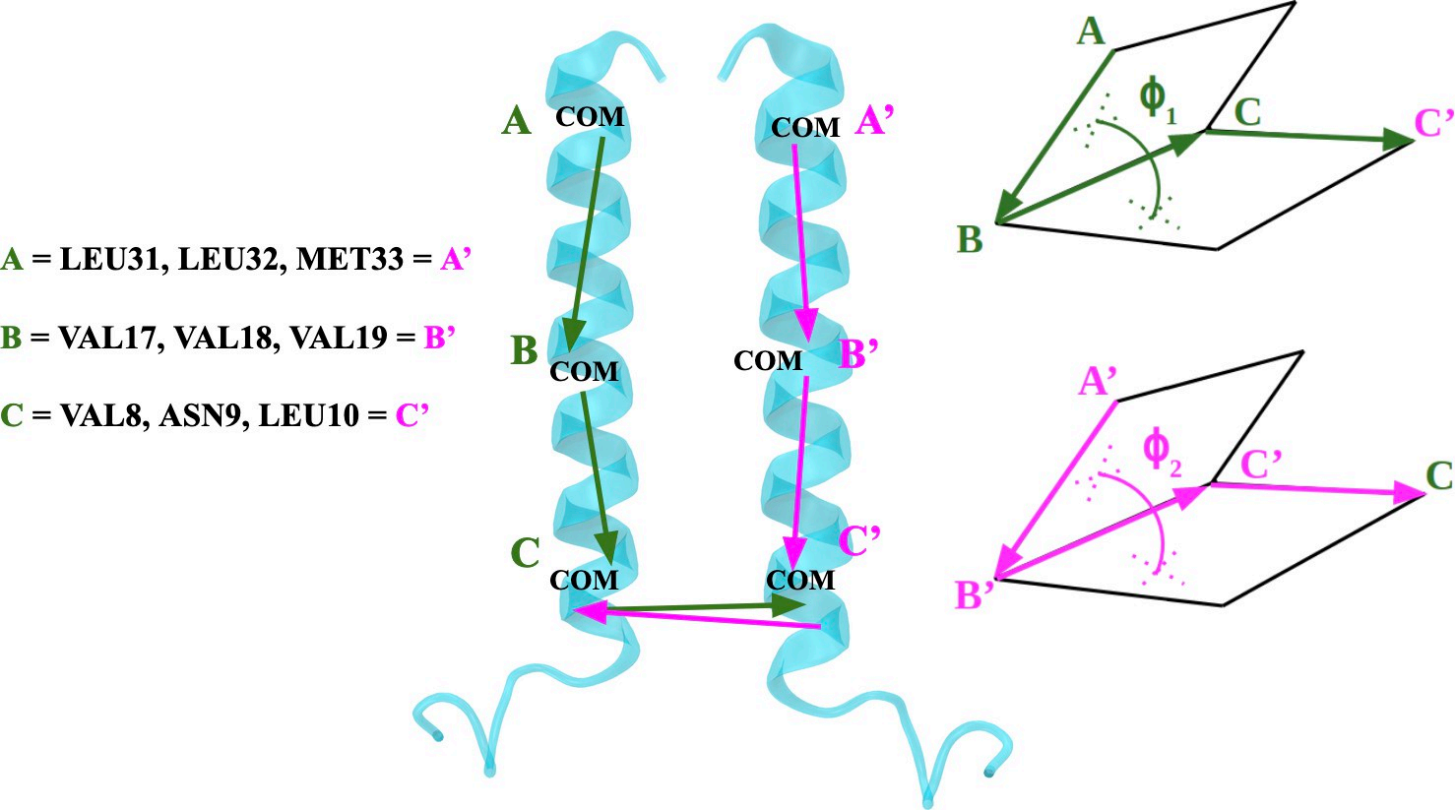
The definition of ϕ_1_ and ϕ_2_ dihedral angles. The centers of mass (COMs) of the upper (A/A’), middle (B/B’), and lower (C/C’) groups of residues were picked as the three points forming each dihedral, while the fourth point (C’/C) belonged to the other monomer. The upper points (A/A’) were defined as the COM of LEU31, LEU32, and MET33 in each SP-C monomer. The middle points (B/B’) were defined as COM of VAL17, VAL18, and VAL19. Finally, the lowest points (C/C’) were defined as COM of VAL8, ASN9, and LEU10.

Free energy calculations employing the coarse-grained MARTINI model were performed using GROMACS 2016 [35] patched with Plumed 2.4.1 [36]. The calculations for free energy were performed using a combination of two-dimensional (2D) umbrella sampling and well-tempered metadynamics using three reaction coordinates: two dihedral angles (ϕ_1_ and ϕ_2_ depicted in Fig 2), which describe the relative orientation of the monomers with respect to each other, and *d*, that is the distance between the centers of mass of the two monomers (see Fig 2 for the definitions of these reaction coordinates). The distance between the centers of mass of the two monomers was used to determine the formation of the dimer, while the dihedral angles allowed efficient sampling of dimerization interfaces. The 2D space spanned by ϕ_1_ and ϕ_2_ was sampled in 10° intervals for each dihedral using a total of 1296 (36 × 36) independent umbrella windows, in which each angle was harmonically restrained to a separate value with a force constant of 500 kJ∙mol^-1^ rad^-2^. This set of windows was later supplemented by 97 additional windows to improve sampling, resulting in a total of 1393 windows. Each window was simulated for 4 μs. The total simulation time of MARTINI simulations was therefore 5.6 ms (1393 × 4 μs). As a side note, the effective time sampled in MARTINI simulations is about 4 times larger than it actually is, as in atomistic simulations, but this conversion factor of 4 is not included in the reported simulation times.

In each window, the distance *d* was sampled continuously using well-tempered metadynamics. The interval within which the metadynamics bias is added was set to 0.5 nm to 4.5 nm and the metadynamics bias was stored on a grid with a spacing of 0.01 nm. Additionally, a half-harmonic restraint with a force constant of 500 kJ mol^-1^ nm^-2^ centered at 5 nm was used to keep *d* within a manageable range. The well-tempered metadynamics bias factor (γ) was set to 50; the width of the Gaussian (σ) hills to 0.05 nm; the initial height of the bias to 0.1 kJ mol^-1^, and the frequency of hill addition to 500 ps.

### Analyses

All post processing and analyses of the CG simulation data were performed using GROMACS 2016 tools [35] and in-house scripts (taking advantage of python packages, such as MDTraj [37]). VMD was used to create figures and to perform visual analyses [38].

All potential of mean force profiles and values, as well as their statistical errors, were estimated by reweighting the free energy simulations as described by Enkavi *et al*. [39] using the free energy simulation data set. Errors were estimated using 100 sets of bootstrap weights generated by the Bayesian block bootstrapping method, in which each free energy window was assigned to a separate block.

**Fig 3:**
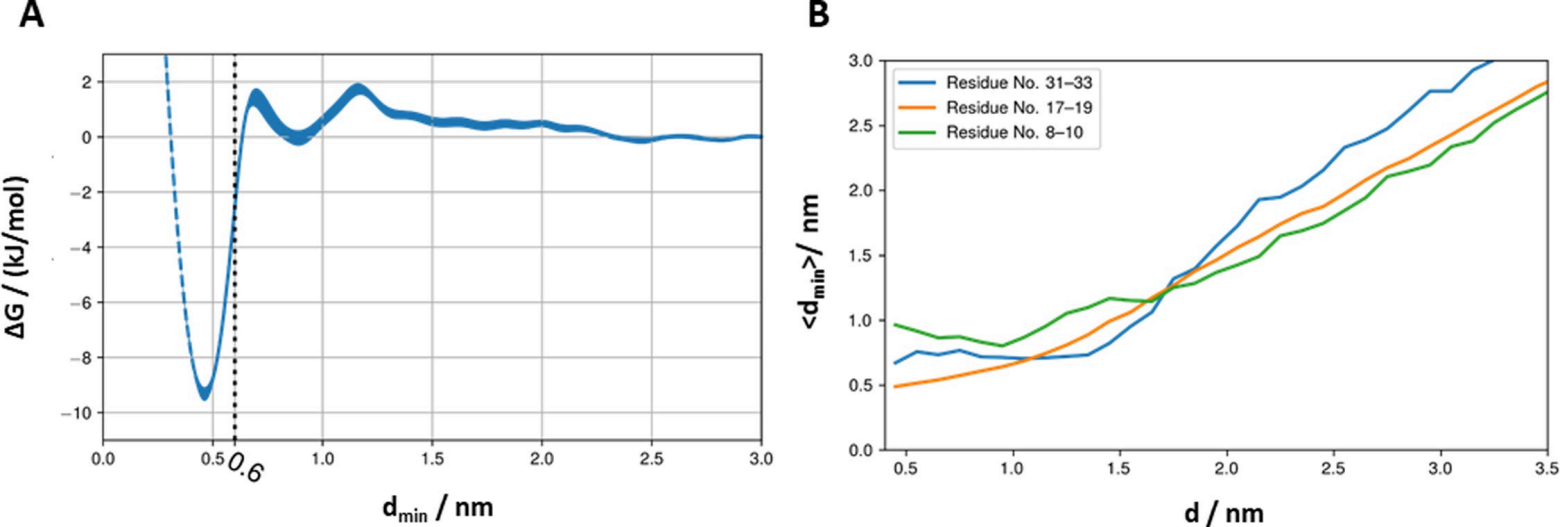
A) The SP-C dimerization free energy as a function of the minimum distance between any two beads belonging to SP-C monomers. Structures with *d*_min_ smaller or equal to 0.60 nm (depicted as black dashed line) are considered the bound dimers. We marked the untapped region of the free energy profile with a blue dashed line. B) The average minimum distance between N-termini (residues 8–10; green line), middles of helices (residues 17–19; orange line), and C-termini (residues 31–33; blue line) of SP-C monomers as a function of the COM-COM distance between SP-C monomers.

Clustering of the dimeric configurations was performed using the agglomerative clustering algorithm implemented in scikit-learn [40] on a subset of protein configurations obtained from the free energy simulations. This subset consists of dimer configurations with a minimum distance (*d*_min_) between the monomers below 0.6 nm. This cutoff distance was chosen based on the boundaries of the basin in Fig 3 to encompass dimer conformations that are more stable than non-interacting monomers. As the distance matrix, the root mean square deviation (RMSD) between all pairs of configurations were used, taking into account that individual monomers are indistinguishable. Note that *d*_min_ and the RMSD matrix are calculated only for the helical segment of the protein (residues 9 to 33) to avoid artefacts due to the terminal flexible loops. Number of clusters was chosen as three based on visualization of the dendrogram. The set of weights associated with the clustered configurations were used in estimation of the free energy values and average properties of each cluster.

### Atomistic simulations

To assess the stability of the dimerization interfaces identified in CG simulations, atomistic simulations of SP-C dimers were performed. All-atom models of the observed (most stable) SP-C dimers were created by fine-graining centroid structures of each cluster obtained in the CG simulations as described above. These low-free energy structures correspond to the *d*_*min*_ values of 0.45 nm, 0.46 nm, and 0.47 nm. Additionally, for comparison, three random high-free energy dimeric structures corresponding to the *d*_*min*_ values of 0.52 nm, 0.57 nm, and 0.58 nm were chosen. Dimer fine-graining was done using the *all-atom converter* tool available in Charmm-GUI [41]. Each all-atom dimer was subsequently embedded in a lipid bilayer composed of 50 mol% DPPC, 25 mol% POPC, 15 mol% POPG, and 10 mol% cholesterol, using the method described elsewhere [32]. These bilayers were solvated, and an appropriate number of counter ions was added to neutralize the system. This resulted in 18 atomistic simulations–three repeats (obtained by three re-insertions of SP-C dimer into lipid bilayer) of three low-free energy dimers, and three repeats (obtained by three re-insertions of SP-C dimer into lipid bilayer) of three high-free energy dimers. After initial energy minimization, four equilibration steps were performed: i) 20 ns of NVT equilibration, ii) 20 ns of NPT equilibration with position restraints on protein heavy atoms, iii) 20 ns of NPT equilibration with position restraints on the backbone atoms, and iv) 100 ns of NVT equilibration with dihedral restraints on the SP-C helix (residues 8–32). Finally, a production run of 1 μs was performed for each atomistic simulation. During the production runs, the systems were simulated in the NPT ensemble at a temperature of 310 K and a pressure of 1 atm. The Nose-Hoover thermostat and the Parrinello-Rahman semi-isotropic barostat with coupling constants of 0.4 and 1.0, respectively, were used [34,42,43]. The simulation time step was 2 fs. The OPLS-AA force field [44,45] was used. All atomistic simulations were performed using GROMACS 2019. PME was used for the treatment of electrostatic interactions. The distance for Coulomb interactions cutoff was set to 1.0 nm, while the van der Waals cutoff was 1.0 nm. The TIP3P water model was used to describe water molecules.

## Results

### Energetics of SP-C homodimer formation

To elucidate the dimerization interface of SP-C thoroughly and to characterize its mechanism, we used a comprehensive set of biased simulations. The free energy calculations were performed using a combination of 2D umbrella sampling and well-tempered metadynamics using three reaction coordinates: two dihedral angles, ϕ_1_ and ϕ_2_ (Fig 2), which describe the relative orientation of the monomers with respect to each other, and *d*, the distance between the centers of mass of the two monomers. The dihedral angles (Fig 2) were defined in the following way: the centers of mass (COMs) of the upper (A, A’), middle (B, B’), and lower (C, C’) groups of residues were picked as the three points of each dihedral, while the fourth point (D, D’) belonged to the other monomer. The upper points (A, A’, see Fig 2) were defined as the COM of LEU31, LEU32, and MET33 in each SP-C monomer. The middle points (B, B’, see Fig 2) were defined as COM of VAL17, VAL18, and VAL19. Finally, the lowest points (C, C’, see Fig 2) were defined as COM of VAL8, ASN9, and LEU10.

The chosen reaction coordinates for the free energy calculations allowed us to sample comprehensively all possible interfaces of SP-C dimers. However, these reaction coordinates are limited in their ability to capture the dimerization process. For example, 2D projection of the free energies on the dihedral angles does not manifest any detectable correlation between the dimeric and monomeric states. To investigate the mechanism and energetics of the dimerization process in detail, we projected the free energy profiles onto other relevant collective variables.

One of those is the minimum distance (*d*_min_) between any two residues belonging to different SP-C monomers as a metric for dimerization. The free energy profile projected onto *d*_min_ (Fig 3A) shows a minimum at 0.45 nm. The free energy difference between the dimeric and monomeric structures of SP-C is about 10.14 kJ/mol. At 310 K, the corresponding thermal energy is about 2.58 kJ/mol. This clearly shows that the SP-C monomers tend to dimerize in the pulmonary surfactant membranes. The profile also reveals another shallow minimum centered at 0.8 nm. This state is approximately equal in free energy to the dissociated monomeric state and taking into consideration the limited space between monomers it likely represents states where monomers are separated by a single lipid molecule before forming a more stable dimer.

Fig 3B shows the mechanism of SP-C homodimer formation. As the minimum distance between the SP-C monomers decreases, all three regions (N-terminus, middle of the helix, and C-terminus) of the SP-C helices come together to form a dimer. However, the C-termini of SP-C monomers come together first and stabilize at the average minimum distance of ~0.7 nm. Subsequently, the middle sections of the helices start to interact together with an average minimum distance of ~0.5 nm. Finally, the N-termini of SP-C monomers come closer together with an average distance of ~1 nm.

All structures where the minimum distance between SP-C monomers is smaller than or equal to 0.60 nm (see the black dashed line in Fig 3A) are considered to be bound dimers, and all subsequent analyses have been performed on the bound dimers only.

### Dimerization Interface and Dimer Conformations

**Fig 4:**
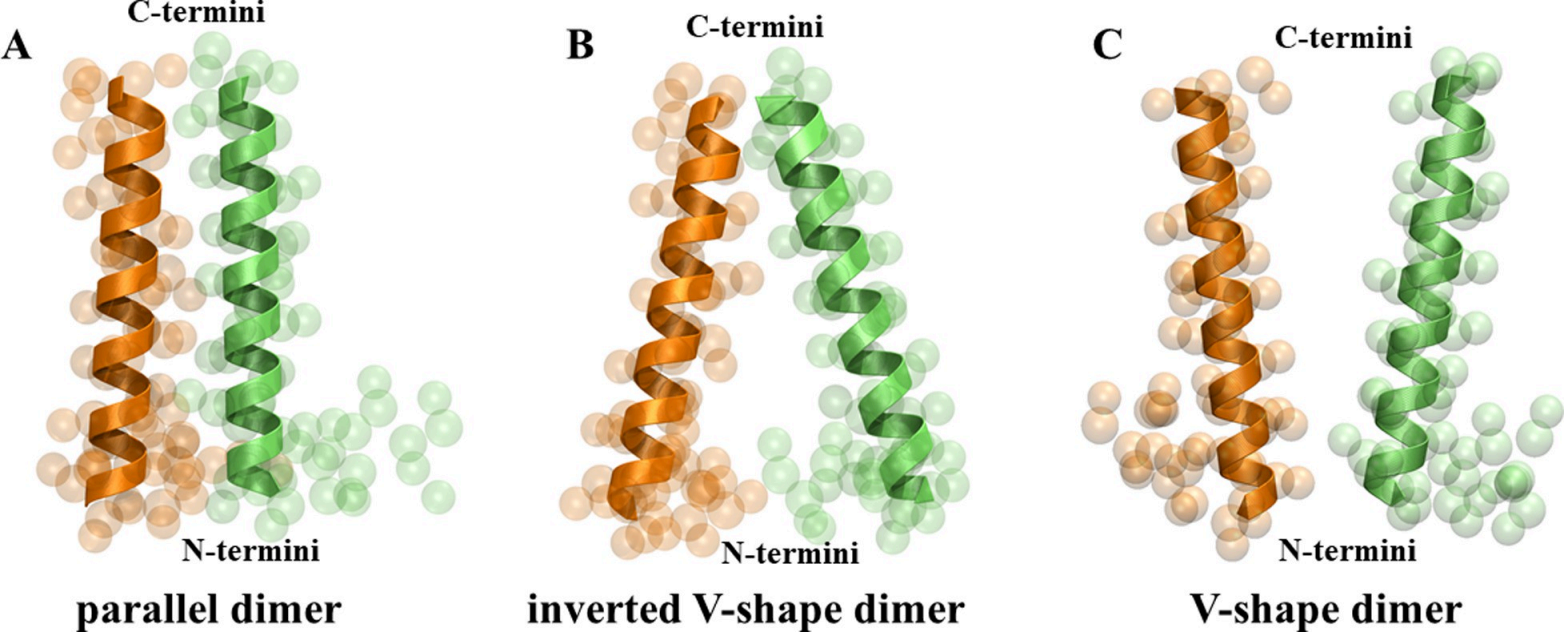
Representative structures (centroids) of three distinctive topologies of the SP-C homodimer ordered by the increasing free energy values: (A) parallel dimer, (B) inverted V-shape dimer, and (C) V-shape dimer. The SP-C monomers involved in the formation of homodimers are colored differently (orange and lime) for clarity.

To get an insight into the formation of SP-C homodimers, we clustered all homodimeric bound structures of SP-C obtained from the free energy calculations using the agglomerative clustering algorithm implemented in scikit-learn, using the RMSD of the helical segment between pairs of configurations as the distance matrix. Representative structures (centroids) of these clusters are presented in Fig 4. This analysis yielded 3 distinctive topologies of SP-C homodimers: *parallel dimer* (Fig 4A), *inverted V-shape dimer* (Fig 4B), and *V-shape dimer* (Fig 4C). Average minimum distance between monomers in each dimer is 0.462 ± 0.000 nm, 0.469 ± 0.001 nm, and 0.495 ± 0.003 nm, for parallel dimer, inverted V-shape dimer, and V-shape dimer, respectively. The stability of the homodimers decreases in the following order: parallel dimer > inverted V-shape dimer > V-shape dimer, as depicted by the free energy: -10.4 ± 0.3 kJ/mol, -5.0 ± 0.4 kJ/mol, and -2.4 ± 0.4 kJ/mol, respectively.

**Fig 5:**
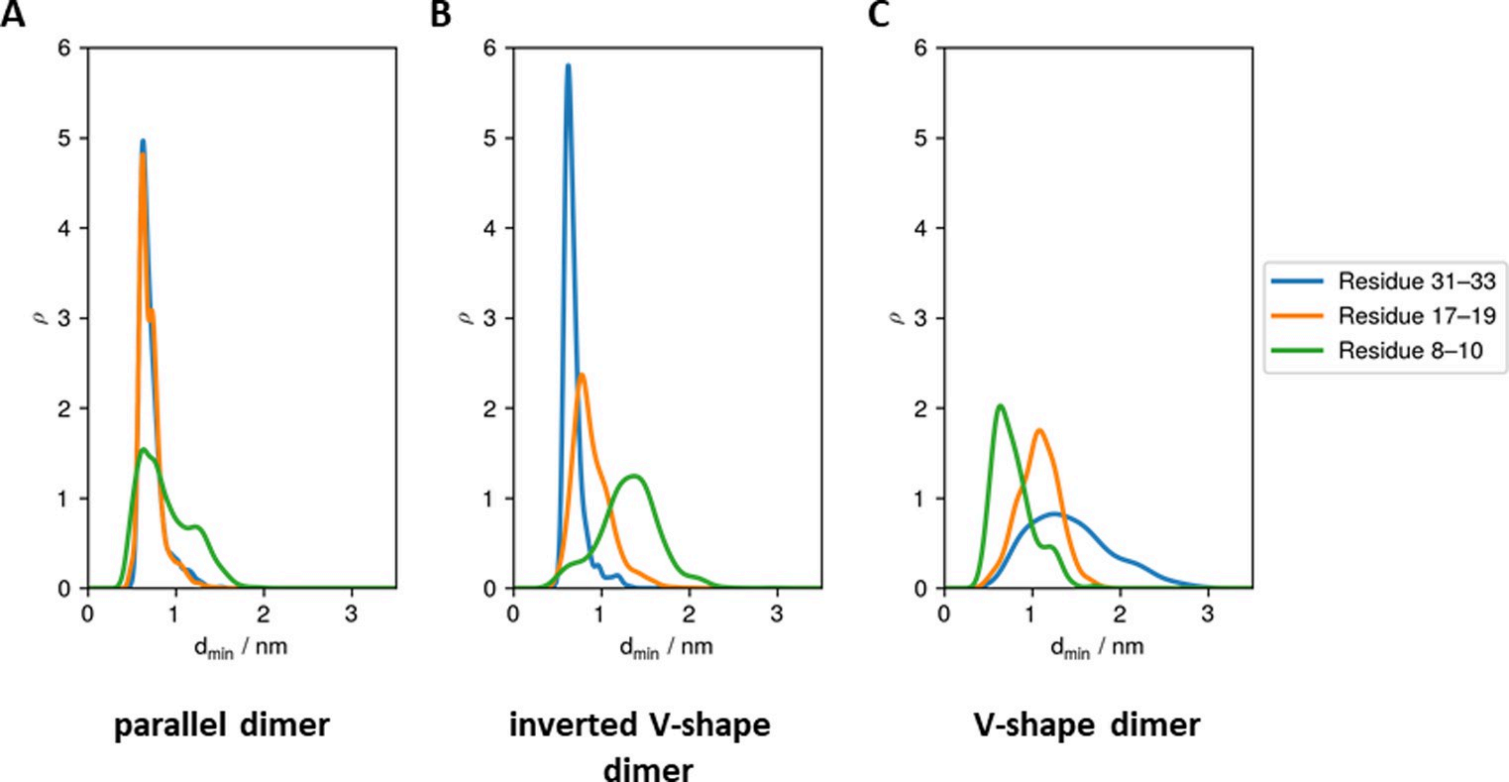
The distributions of the minimum distances between N-termini (green line), middles of helices (orange line), and C-termini (blue line) of SP-C monomers in each dimer topology: A) parallel dimer, B) inverted V-shape dimer, and C) V-shape dimer.

To characterize each topology, we plot the distributions of the minimum distance between N-termini, middles of helices, and C-termini of SP-C monomers in each dimer topology. As shown in Fig 5, the minimum distances of all three regions (N-terminus, middle of the helix, and C-terminus) are the shortest for the parallel dimer (Fig 5A), with the maxima of the distributions being 0.63 nm, 0.63 nm, and 0.62 nm, for N-termini, middles of helices, and C-termini, respectively. The inverted V-shape dimer (Fig 5B) has a similar minimum distance of the C-termini (with the maximum of the distribution being 0.62 nm), however both the middles of the helices and N-termini are shifted towards higher values of minimum distance (with the maximum of the distribution being 0.78 nm and 1.37 nm, respectively). In the case of V-shape dimer (Fig 5C), the distance between N-termini is the shortest followed by the middles of the helices and C-termini, with the maxima of the distribution being 1.25 nm, 1.08 nm, and 0.64 nm, respectively.

**Fig 6:**
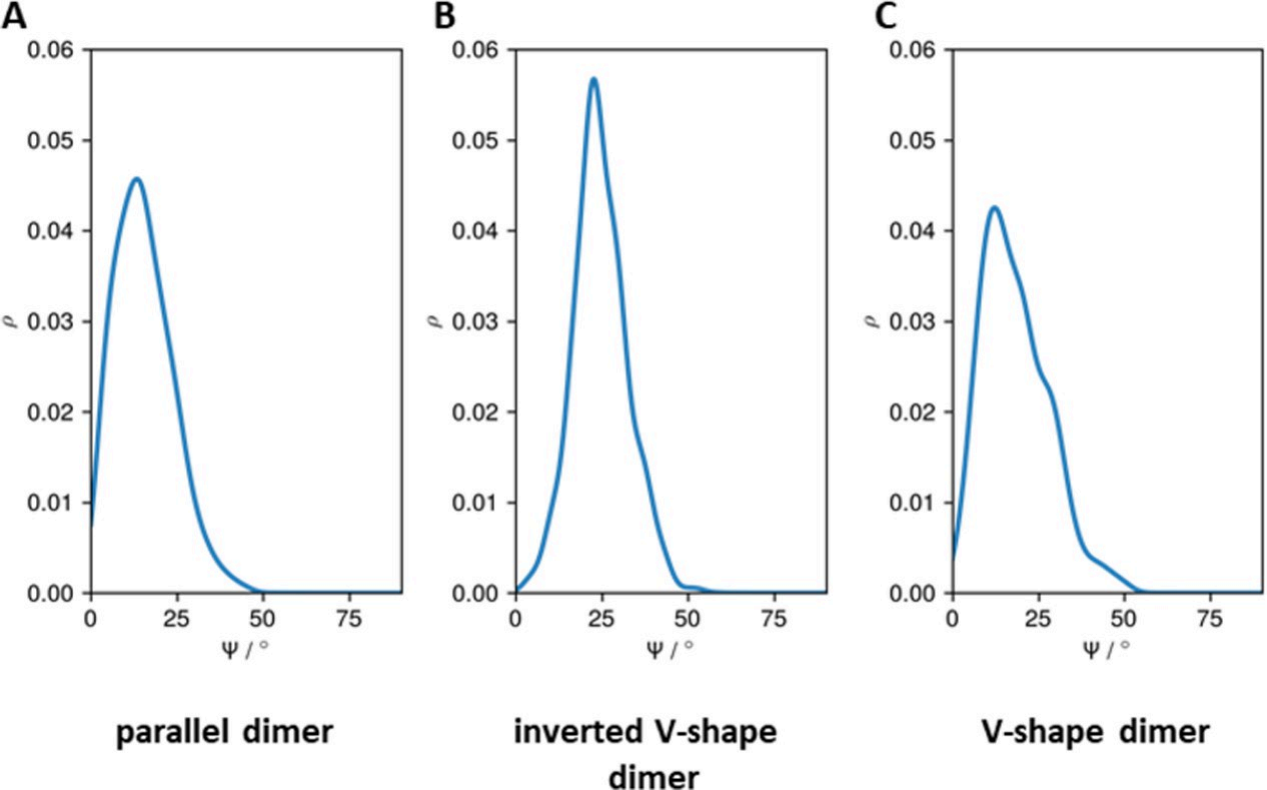
The distributions of the crossing angles (Ψ) between the helices forming the homodimer in each dimer topology: A) parallel dimer, B) inverted V-shape dimer, and C) V-shape dimer.

To further characterize the topological differences between dimers, the crossing angle (Ψ) between the helices forming the homodimer was calculated. The crossing angle was defined as the angle between the helical axes of the two monomers. The helical axis was defined as the vector from the center of mass of the backbone (BB) atoms of residues 8–11 to that of residues 30–33. In the parallel dimer (Fig 6A), the dimer adopts conformations where the crossing angle is small, with the average crossing angle being 15.4°. The inverted V-shape dimer (Fig 6B) adopts conformations with higher values of the crossing angle, with an average being 24.6°. Interestingly, the average crossing angle in the V-shape dimer (Fig 6C) is only 18.3°.

**Fig 7:**
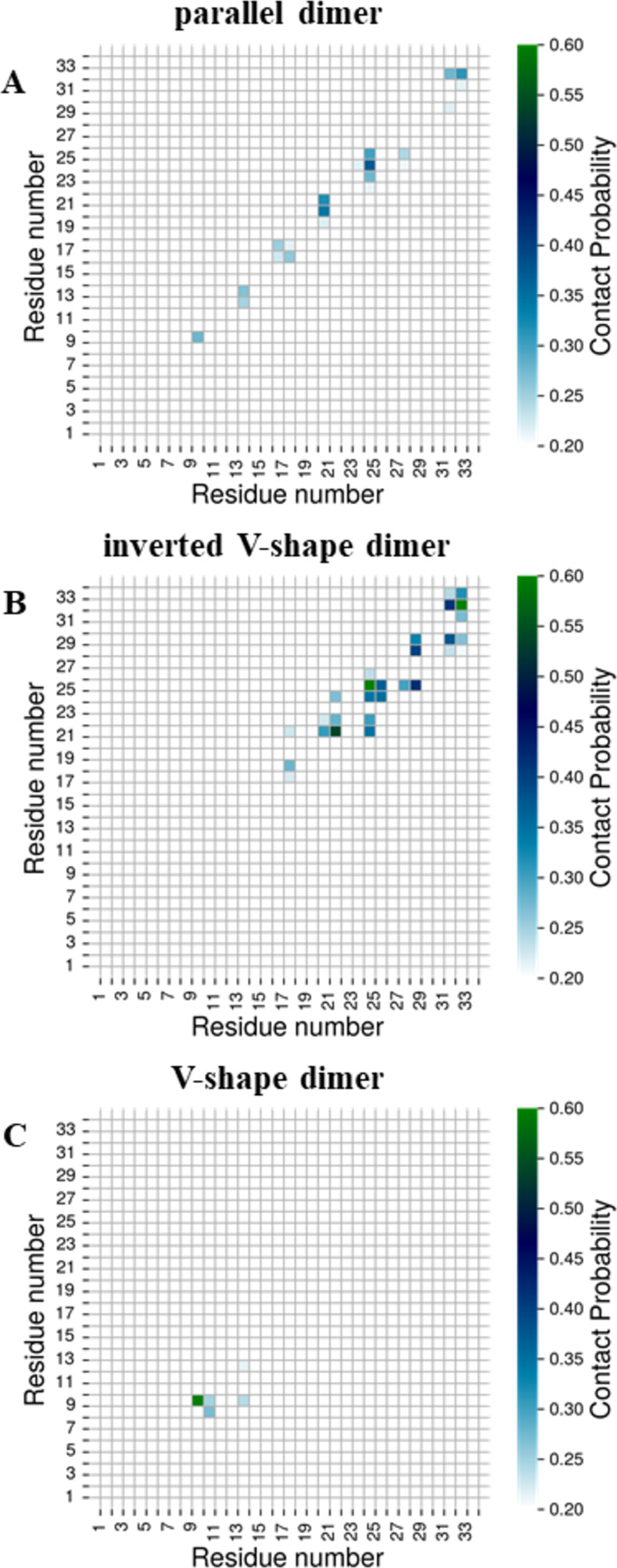
Contact probability maps for each pair of residues in the SP-C homodimers averaged over all homodimers belonging to the given dimer topology. Contact probabilities equal to one correspond to the situation where given residues are in contact in all homodimers belonging to this cluster topology. Two residues were considered to be in contact, if any of their beads were closer than 6 Å. Only contact probabilities higher than 0.20 are shown for clarity.

To identify the residues that are actually involved in the dimerization interface in each dimer topology, contact maps between each residue in SP-C dimers were calculated. Fig 7 shows the averaged contact probability maps of each pair of residues in the SP-C homodimers belonging to different dimer topologies.

In the lowest free energy dimer (parallel dimer, Fig 7A), corresponding to the most stable case, the residues L10, L14, L18, V21/L22, V25/V26, G29, and M33 in each chain, are showing a high propensity to interact with each other. In this conformation, V21/L22 and V25/V26 play the most important role (the highest propensity to interact). In the inverted V-shape dimer (Fig 7B), residues L22, V26, G29, and M33 in each chain interact the most with each other. Although the crossing angle in the V-shape dimer is similar to the angle in the parallel dimer, the dimerization interface in this topology (Fig 7C) is completely different–the highest propensity to interact with each other have L10 residues.

### Structure refinement by atomistic simulations

An ideal computational approach to explore the dimerization of SP-C monomers would be atomistic molecular dynamics. However, in practice this approach is not sufficiently feasible. As we mentioned above, the total time simulated in this project was larger than 5 milliseconds, and if the typical speed-up factor of 4 associated with MARTINI simulations would be accounted for (due to the dynamics that in CG MARTINI simulations take place faster than in atomistic simulation models), atomistic simulations of this project would have required >20 milliseconds, which was not possible. However, given that the sampling of our CG simulations is based on 5 millisecond simulations, and the dynamics in atomic-level simulation models would evolve more slowly, it is not very clear that achieving a similar level of sampling in atomic-level simulations would be sufficiently feasible. Nonetheless, to confirm that the predictions of the present CG simulations are reasonable, we studied the stability of the key dimer structures through all-atom simulations.

The SP-C dimers found through CG simulations were fine-grained to atomistic resolution. The low-free energy (the most stable) structures correspond to the *d*_*min*_ values of 0.45 nm, 0.46 nm, and 0.47 nm. Additionally, for comparison, three random high-free energy (the least stable) dimeric structures corresponding to the *d*_*min*_ values of 0.52 nm, 0.57 nm, and 0.58 nm were chosen. After embedding the dimer structures into lipid bilayers and subsequent equilibration, 18 atomistic production simulations–three repeats of three low-free energy dimers, and three repeats of three high-free energy dimers–were performed for 1 μs each. As a key figure of merit, the stability of the dimers was assessed by considering the minimum distance between monomers in each dimer as a function of simulation time. These data revealed that low-free energy dimers found in the CG simulations are indeed stable in atomistic simulations, while the high-free energy dimers were found to be unstable in atomistic resolution simulations, thus confirming the key results of CG simulations.

## Discussion

Our multi-scale molecular dynamics simulations show the mechanism and energetics of SP-C dimerization in lipid bilayers. The simulations revealed three distinct topologies of the SP-C homodimer. The lowest free energy topology, the parallel dimer, corresponding to the most stable structure observed, is characterized by low values of the minimum distances in all three regions (N-terminus, middle of the helix, and C-terminus) and the lowest average value of the crossing angle. The second lowest free energy topology, the inverted V-shape dimer, has a similar (to a parallel dimer) arrangement in the C-termini region but is substantially different in the middle and N-termini regions, and it also adopts much higher values of the crossing angle. The V-shape dimer represents the highest free energy (corresponding to the least stable) dimeric topology revealed by this study. This dimer adopts on average similar crossing angle values as the parallel dimer but differs substantially in the C-termini and middle region of SP-C helices.

The two lowest free energy topologies, the parallel dimer and the inverted V-shape dimer, are characterized by similar minimum distances in the C-termini region, giving rise to a similar dimerization interface (comprised of residues V21/L22, V25/V26, G29, and M33) in this region. However, the minimum distances in the middle of helices and N-termini regions as well as substantially different crossing angles are responsible for the lack of the interacting residues in the middle and N-termini regions of the inverted V-shape dimer, contrary to the parallel dimer. Interestingly, the dimerization interface in the V-shape dimer differs substantially from that in the parallel dimer despite similar average crossing angles in both topologies. This suggests that both the crossing angle and the arrangement in N-termini and C-termini regions must be similar for comparable dimerization interfaces. Dimerization at the N-terminal region of the helices is energetically less favorable when compared to dimerization at the center or the C-terminal region. This can be also seen in the mechanism of dimerization. C-terminal is the region where the initial contacts take place, followed by the coupling at the center of the helices. However, the N-terminal region resists dimerization and close contact. This is likely due to the positively charged ARG2. Moreover, the two PRO residues (PRO4 and PRO7) might be restricting favorable conformations and rendering its dimerization entropically unfavorable.

**Fig 8:**
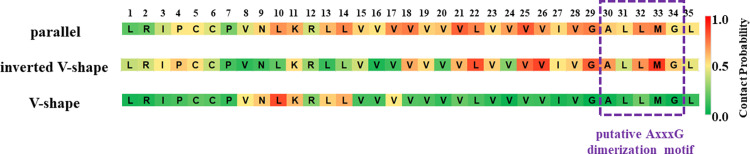
Contact probabilities of SP-C residues for all dimer topologies identified in this study. The purple dashed line depicts the position of the putative AxxxG motif as reported by Kairys *et al*. [23].

Although SP-C does not contain a classical dimerization GxxxG motif, the AxxxG motif is present in its structure and was suggested as a putative dimerization motive by Kairys *et al*. [23]. Fig 8 shows the contact probabilities of all SP-C residues for all dimer topologies identified in this study. Our data show that the putative AxxxG motif does not participate in the SP-C dimerization. For the lowest free energy dimers, the parallel dimer and the inverted V-shape dimer, the region comprised of residues V21/L22, V25/V26, G29, and M33 seems to be responsible for the SP-C dimerization in the pulmonary surfactant bilayers. This region falls into the V^21^xxxVxxxGxxxM^33^ motif, with x being a small residue.

It has been proposed that a rigid inverted V-shape structure of SP-C, followed by clustering of protein dimers, could sustain progressive protein-promoted curvature of surfactant membranes preceding budding of lipid/protein nanovesicles [26,46]. This budding could be important for a depuration of surfactant from the less surface-active lipids, which could be then targeted to pneumocytes or macrophages for recycling or catabolism. Our simulation experiments therefore confirm the potential of SP-C to form such inverted cone-shaped dimeric structures, which could be particularly enriched in defined surfactant lipid compositions. The identification here of the key residues to drive formation of stable dimers will allow testing to what extent targeted mutations prevent SP-C-promoted membrane microvesiculization, and the potential connection of this activity with the role of SP-C in surfactant alveolar homeostasis.

Based on experimental data, it is possible that SP-C forms oligomeric structures larger than dimers, either with the SP-B protein or by itself [47]. Our simulations suggest that SP-C has several dimerization interfaces, and the existence of the three dimerization interfaces observed in this work might be involved in the formation of higher oligomeric structures.

## Conclusions

The dimerization of the surfactant protein C emerges as an important step for the understanding of its biological function in the pulmonary surfactant. Although the dimeric structures of surfactant protein C have not yet been resolved experimentally by NMR, X-ray, or cryo-EM techniques, our computational approach, based on the 2-dimensional umbrella sampling and well-tempered metadynamics, revealed three topologically different homodimeric states of SP-C. We characterized the structural properties and dimeric interfaces of each of the dimeric states, and we found that the putative dimerization motif, the AxxxG motif, is not present in the dimerization interface of SP-C. Instead, a substantially larger region of the SP-C protein, namely the V^21^xxxVxxxGxxxM^33^ motif, could play a clearer role in SP-C dimerization. Our computational findings can be tested experimentally by performing mutagenesis studies on the key residues (e.g., V21/L22, V25/V26, G29, M33) in the V^21^xxxVxxxGxxxM^33^ region of SP-C.
